# Connexins and Gap Junctions in Cancer of the Urinary Tract

**DOI:** 10.3390/cancers11050704

**Published:** 2019-05-22

**Authors:** Thomas Tschernig

**Affiliations:** Institute for Anatomy and Cell Biology, Medical Faculty, Saarland University, 66421 Homburg/Saar, Germany; thomas.tschernig@uks.eu; Tel.: +49-6841-1626100; Fax: +49-6841-1626121

**Keywords:** connexins, gap junctions, intercellular communication, human urinary tract, cancer, carcinogenesis

## Abstract

This review focuses on connexins and nexus or gap junctions in the genesis, progression, and therapy of carcinomas of the human urinary tract. Some decades ago, the idea was born that gap junctional intercellular communication might prevent both the onset and the progression of cancer. Later evidence indicated that, on the contrary, synthesis and the presence of connexins as a prerequisite for gap junctional intercellular communication might promote the occurrence of cancer and metastases. The research history of urinary bladder cancer is a good example of the development of scientific perception. So far, the role of gap junctional intercellular communication in carcinogenesis and cancer progression, as well as in therapeutical approaches, remains unclear.

## 1. Introduction

**Figure 1:**
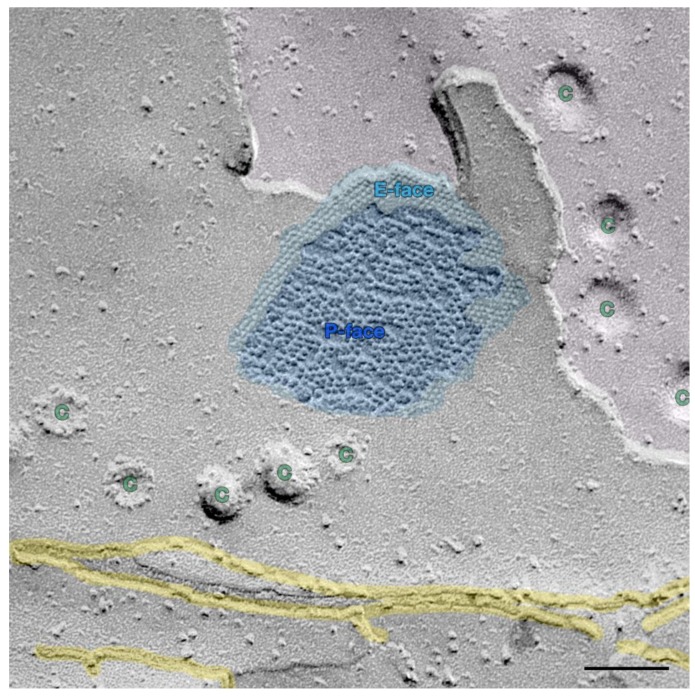
Freeze-fracture image of a gap junction in the myelin sheath of an optical nerve axon (mouse), consisting of P-face (the P-face, close to protoplasm, is the inner lamella of the plasma membrane viewed from outside the cell; deep blue) particles and E-face (the E-face, close to the extracellular space, is the outer lamella of the plasma membrane viewed as if from within the cell; pale blue) pits, surrounded by tight junctions (yellow) and caveolae (green ‘C’s). Underlying myelin layer in pale red. Scale bar depicts 0.1 µm.

Gap junctions facilitate intercellular communication and regulate proliferation and differentiation [1]. Since gap junctional intercellular communication (GJIC) is necessary to mediate contact inhibition in growing cells and tissues, inhibited GJIC might reduce growth control and differentiation [2] and is a hallmark of epithelial-derived cancer cells [3]. James Trosko stated that epigenetic tumor promoters and activated oncogenes can block gap junction function and yield insights into the complex [2]. Gap junctions are formed by two connexons or hemichannels of neighboring cells. These hemichannels are made up of six subunits of connexins. At total of 21 different connexins are known in humans [4,5,6] and these are named according to their approximate molecular weight. The typical morphology of a gap junction is shown in Figure 1. To achieve such photographs of gap junction ultra-structures with transmission electron microscopy, a special technique, namely the freeze-fracture technique, is necessary [7,8]. Gap junctions are channels which bridge two separate cells, enablingions, second messenger signals, and other molecules (<1kD) to reach the cytoplasm of adjacent cells [9,10]. A prime example is the intercalated discs in the heart muscle, where connexins form gap junctions and enable the distribution of calcium to spread the excitation from pace maker nodes to all parts of the heart [11]. In their general contribution to intercellular communication, connexins and gap junctions also regulate proliferation and cellular differentiation [1,12].

The contribution of gap junctions and nexus to intercellular communication has been the subject of heated discussions in the field of carcinogenesis. Highlighted are the interesting overviews presented by Aasen et al. and Graham and colleagues [13,14]. Observations of connexins and gap junctions in tumors are abundant, and their possible role, both in carcinogenesis and as targets for cancer therapy, are broadly discussed [4,15]. The following section focuses on findings and hypotheses relating to the different parts of the urinary tract and its carcinomas. The urinary tract consists of organs which produce and control urine: The kidneys, the ureter, the urinary bladder, and the urethra. Closely related epithelial organs, such as prostate and seminal vesicles, which are necessary for reproduction, are also included in this context. In general, gap junctions have rarely been reported in the healthy human urinary tract at all; these reports will be summarized at the end of the review. Most information is available for the human urinary bladder: Gap junctions, mainly connexin 43, were found in the smooth muscle cells of the detrusor muscle of the urinary bladder [16]. Connexin 43 has additionally been detected in interstitial cells, which are located close to the epithelium [17]. The epithelium of the human urinary tract, which is called the urothelium, was found to be negative for connexins 40, 43, and 45. In contrast, connexin 43 has been detected in the urothelial layer of the bovine urinary bladder and its cancer [18].

## 2. Kidney and Renal Cell Carcinoma

While data sets on the expression of different connexins within kidney epithelial cells—mostly compiled in cell lines—are limited, data on gap junctions within kidney tissues are even rarer. Gap junctions have been reported on the lateral membranes of cells of proximal tubules of human fetal kidneys [19]. Wilgenbus and colleagues have presented data on the expression of Cx26, Cx32, and Cx43 in normal kidney tissue and in carcinomas of kidney, prostate, and testis, but also of liver, breast, esophagus, skin, lung, and uterus [20]. In the kidney, they found immunoreactivity for Cx26 and Cx43 in the epithelium of proximal tubules. A subtle signal for Cx43 was detected in three renal cell carcinomas in the same study. To our knowledge, no data are available on gap junctions in renal carcinoma. As mentioned above, there are more studies which have analyzed the expression of connexins in cultured human tubule cells, such as, for instance, in cells of the proximal tubule [21]. In that study, “aggregates thought to represent gap junctions” [21], were presented in images of freeze-fracture replicas and cells were shown to be functionally connected by electrophysiology. In kidney cell lines, gap junctional intercellular communication (GJIC) was mostly investigated by dye transfer assays, such as scratch labelling, e.g., by Noguchi et al. In one renal cancer cell line, they detected a decreased expression of Cx43 and correspondingly reduced GJIC, whereas in another line, no Cx43 expression at all, corresponding to the complete absence of GJIC, was measurable [22]. The same group reported that all-trans retinoic acid enhanced GJIC between carcinogen-treated renal epithelial cells [23]. Another interesting member of the connexin family is connexin 32 [24]. Connexin 32 was found to be involved in a number of inherited neuropathies and especially in the Charcot-Marie-Tooth disease [25,26]. In addition, a role in tumorigenesis has been described for this connexin [27]. The group led by Yano published several studies on the role of connexin 32 in renal cell carcinoma and claimed that connexin 32 regulates “proliferation, invasion, and metastasis” in renal cell carcinoma [28,29]. A more recent report demonstrated that induction of the connexin 32 gene in human renal carcinoma cells enhanced the vinblastine-induced cytotoxicity in human renal carcinoma cells [30].

## 3. Renal Pelvis, Ureter, and Urethra and Their Carcinomas

It is difficult to find specific data for the renal pelvis, the ureter, and the urethra. Connexin 26 has been detected in human urothelium of the urinary bladder using immunohistochemistry on paraffin sections with limited expression in the basal layer. It is not unlikely that this is also true for the urothelium in the renal pelvis, the ureter, and the urethra [31]. One report investigated urethral tissue of rats and sheep [32]. The authors found a positive immunofluorescence for connexin 43 in the urethral urothelium of sheep but not in that of rats. In contrast, the smooth muscle layer was positive for connexin 43 in both species. In the same study a weaker signal was found for connexin 37 in the smooth muscle cells. As far as the authors are aware, no information is available on gap junctions in the normal urothelium of the upper urinary tract and its tumors. Cancer of the upper urinary tract is rare but has been described [33,34]. A main risk factor for urothelial carcinomas and small cell neuroendocrine carcinomas in the urinary bladder and in the urinary tract in general is tobacco smoking or exposure to tobacco smoke, respectively [35]. The assumption seems feasible that many findings described for urinary bladder carcinomas are also true for the urinary tract in general, including the renal pelvis and the ureter, dealt with in the following chapter.

## 4. Urinary Bladder and Urinary Bladder Carcinoma

**Figure 2:**
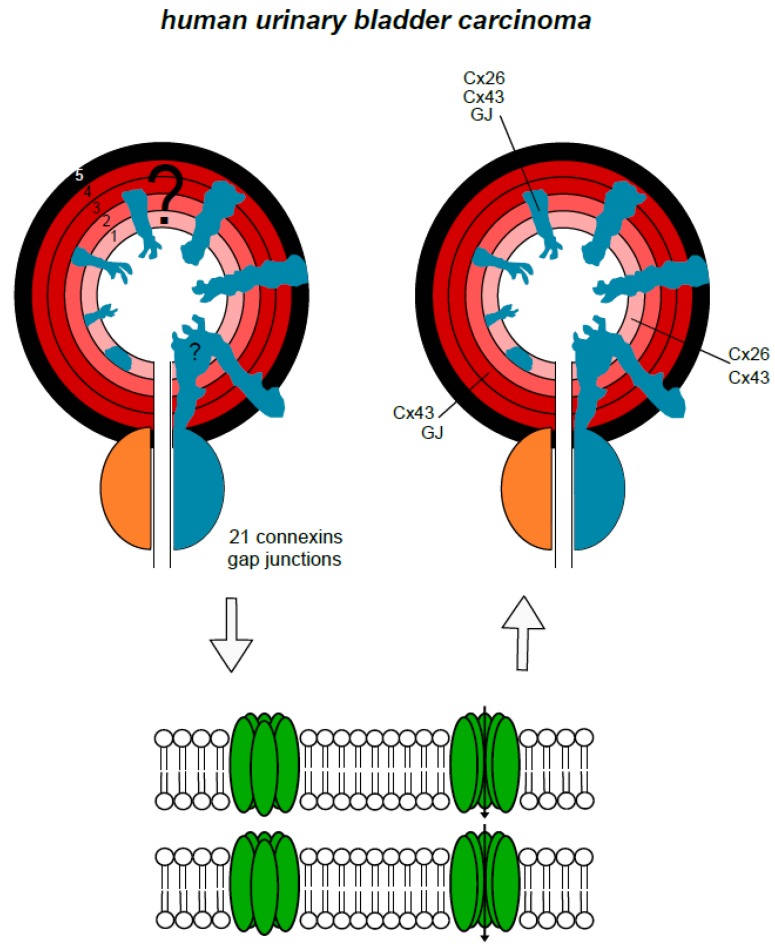
Schematic drawing of the layers and tumor distribution in the human urinary bladder and its cancer. The question marks relating to the expression of connexins in the various layers on the left side are answered, and, on the right side, are answered where possible. Finally, there are indications that connexons and gap junctions are present in the detrusor muscle and adenocarcinoma of the human urinary bladder. (orange = healthy prostate, blue carcinoma, 1 = urothelium, 2 = lamina propria and to some extent submucosa, 3 = detrusor muscle inner layer, 4 = detrusor muscle outer layer, 5 = adventitia).

**Figure 3:**
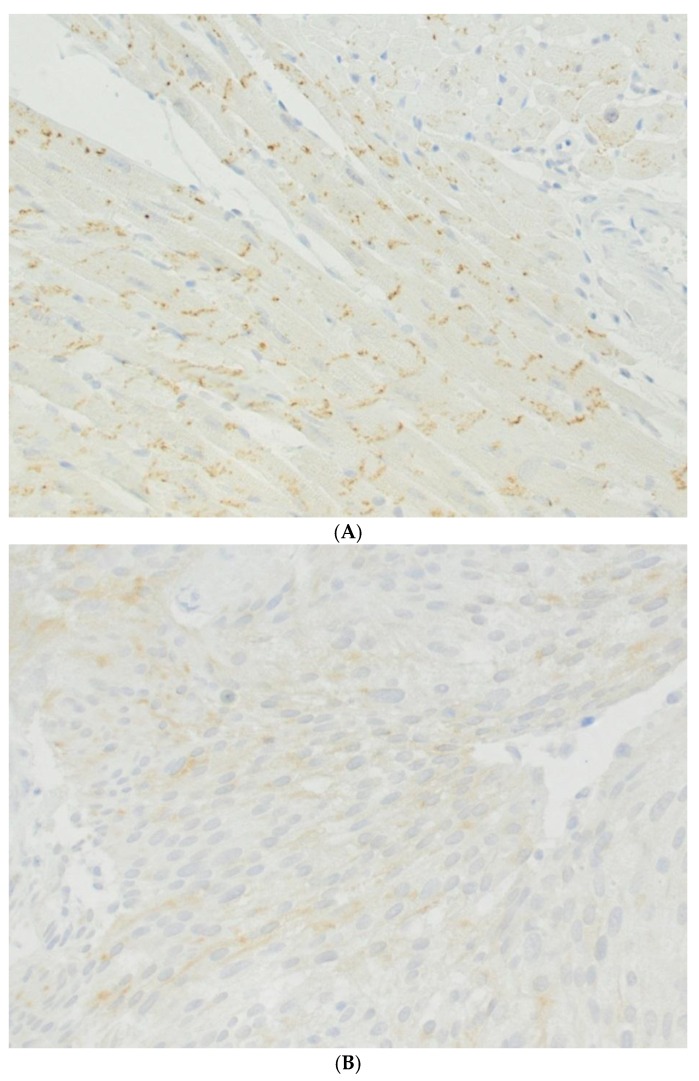
Representative microphotographs show the specific labeling of connexin 43 in the heart (**A**). The intercalated disks which express connexin 43 are labeled with a brown dye. A weaker but clear expression of connexin 43 can be observed on a section of a human urinary bladder cancer (**B**).

Urinary bladder cancer is among the 15 most common cancers worldwide. It has a higher incidence in Europe, North America, and Australia and a lower incidence in the Far Eastern countries [36]. More than 400,000 new cases were counted in 2012. Urinary bladder cancer is the 5th most commonly diagnosed cancer in Europe. Its incidence increases with age and it is most common between the 60th and 70th year of life. More men than women are affected. Exposure to tobacco smoke is the main risk factor [36,37]. In the human bladder gap junctions, connexin 43 has been found in cells of the detrusor muscle [16]. The gap junction protein connexin 43 has also been detected in so-called interstitial cells beneath the urothelium, and human urothelium was found to be a negative for connexin 40, 43, and 45 [17]. In contrast, connexin 43 has been detected in the bovine urothelium and partly in its tumors [18]. Interestingly, limited expression of connexin 26 has been detected in human urothelium [31]. That report also revealed an altered, weakened expression of connexin 26 in bladder tumors. An overview on the occurrence of gap junction proteins in the different anatomical layers of the normal human urinary bladder and its carcinomas is presented in a schematic drawing (Figure 2). A possible role of gap junctions and connexins in carcinogenesis was discussed for several years [38,39]: The hypothesis was that a loss of connexins and reduced gap junctional intercellular communication correlated to malignancy and the induction of connexins and gap junctions should therefore be beneficial to patients suffering from urothelial carcinoma. In contrast, a converse theory is that enhanced gap junctional intercellular communication or increased expression of connexins promotes urinary bladder carcinogenesis [40]. The expression of connexins in human bladder cancer has been investigated previously [31,41,42]. The investigations of Gee et al. showed the expression of connexin 26 in urinary bladder cancer tissue and cells [31]. A further report by Gee et al. dealt with the treatment of this cancer by deleting the expression of connexin 26 using a gene therapy approach [43]. In the study conducted by Comberg et al., the synthesis of a range of connexins (26, 29, 32, 36, and 43) was investigated using immunohistochemistry on tissue samples of human urothelial carcinoma from nine patients with urothelial carcinoma (5 males, 4 females). A major advantage of an approach using immunohistochemistry is that specific labelling can be assigned to tumor cells or, if applicable, to other tissues, such as interstitial cells, fibrocytes, or muscle cells. Slides were immune-stained with polyclonal antibodies for connexins 26, 29, 32, 36, and 43. Connexin 26 was found in the tumor cells of two of the nine patients. The connexins 29, 32, and 36 were not found in the tumor cells at all. In contrast, connexin 43 was detected in the tumor cells of 8 of the nine patients. It can be concluded from the results in this report that connexins 29, 32, and 36 are unlikely to play a role in human urothelial carcinoma. The main result of this study was that connexin 43 was frequently found in human urothelial carcinomas. Examples of connexin 43 staining using immunohistochemistry was presented showing the typical intercalating discs in tissue of the heart muscle (Figure 3A) and in tissue of a urinary bladder carcinoma (Figure 3B). This supported data from another report on the synthesis or expression of connexin 43 in urinary bladder carcinomas [42]. These authors found frequent expression levels of connexin 43 in (non-muscular) invasive bladder cancer (NMIBC). In addition, high expression levels were associated with a poor prognosis. They even proposed a routine assessment of connexin 43 expression to identify high-risk NMIBC. Since normal human urothelium does not produce connexin 43, the presence of connexin 43 may be tumor-specific or at least somehow tumor-associated. More than one decade ago it was assumed that a loss of connexin 43 led to malignancy and that it might, therefore, be a target for genetic therapeutical approaches [38,39]. As already mentioned, the more recent interpretation of its role is exactly the opposite, i.e., connexin 43 expression promotes carcinogenesis in urinary bladder carcinoma [44]. Connexin 43 enhances the adherence of tumor cells to the stroma as well as the migration and, probably, the dissemination of cancer cells. Interestingly, one of the early evaluations did actually also postulate that “increased gap junctional intercellular communication capacity or increased connexin(s) expression” increased rat bladder carcinogenesis, as tested in cell lines and tumors [40]. The high prevalence of the expression of connexin 43 in the studies of Poyet et al. and Comberg et al. support the latter view [41,42]: The synthesis of connexin 43 in urothelial cancer cells is more likely to be a co-factor in the genesis and growth of urothelial carcinomas than a physiological or protecting factor. This view is supported by recent findings in prostate cancer, where connexins were found to play a role in the dissemination of cancer cells [45]. Another interesting question is the formation of gap junctions in the human urinary bladder and human urinary bladder cancer. With the exception of indirect findings, using calcium flux experiments [46], there are only two publications to be found which present gap junctions, using morphological techniques, such as electron microscopy. One is the report of John et al. who found “small and irregularly shaped” gap junctions in the detrusor muscle of a non-obstructed human bladder using freeze-fracture studies [16]. Freeze-fracture studies are a useful tool in the identification of gap junctions. However, a combination with immunolabelling would be much more efficient as it would enable the visualization of the molecular assembling of cancer-associated gap junctions [8]. The second is an older report by Alroy et al. who found “few and small” gap junctions within primary adenocarcinoma of the human urinary bladder [47]. As can be seen, the role of gap junctions in the context of human urinary bladder cancer is difficult to assess and offers a wide and open field for further research.

## 5. Prostate and Seminal Vesicles and Their Carcinomas

A recent review described connexins as important players in the dissemination of prostate cancer cells [45]. Are there connexins and gap junctions in healthy prostate tissue? The expression of connexin 32 was detected in the human prostatic epithelium [48]. In the same study, a dye transfer was observed between cells of the normal human prostatic epithelium and those of prostate cancer cell lines suggesting gap-junctional communication in both normal and malignant prostate cells. However, the dye transfer was reduced in the malignant cells and not present in certain malignant cells. This observation correlates with an observation made in a study analyzing tissues of prostate cancers. Here, connexin 43 expression was reduced or even missing in prostate cancer tissues [49]. The same study reported that, in contrast, an overwhelmingly high expression of connexin 43 was found in prostate tissue of patients suffering from benign prostate hyperplasia. This is an interesting finding, since an expression of connexin 43 is not reported within the normal prostatic epithelium and is strongly reduced in prostatic carcinomas. The assumption seems feasible that this indicates a continuum in the biology of prostatic epithelium from differentiation over hyperplasia to de-differentiation and, finally, to cancer. The increased expression in hyperplasia might be an epiphenomenon but, on the other hand, it is more likely that connexin 43 is required in tissues with accelerated cell proliferation [50,51]. The seminal vesicles are often invaded by carcinomas, such as the prostate carcinoma. The primary seminal vesicle carcinoma is a rare disease [52]. Interestingly, the expression of connexins in human seminal vesicles has not been investigated so far, but connexin 32 has been found in the seminal vesicles of adult Sprague Dawley rats [53].

## 6. Urinary Tract Carcinomas, Related Toxic Agents and Connexons, Gap Junctions, and Gap Junctional Intercellular Communication

It is well known that, whether in a personal or an occupational environment, exposure to toxic agents, such as tobacco smoke and its ingredients, arsenic, cadmium, and aromatic amines, and further substances, promote the development of carcinomas in the urinary tract—sometimes after a couple of years, or even decades [37]. Do these agents have any influence on the structure and function of gap junctions? To start with: Tobacco smoke. James Trosko et al. reported the inhibition of GJIC by 1-methylanthracene, a component of tobacco smoke [3]. A scrape loading/dye transfer technique, which had been developed earlier by the same group, was applied to a cell line derived from rat liver (WB-F344) [54]. They found that a distinct phospholipase C was important for the inhibition of GJIC and non-mitogen-activated protein kinases, as previously assumed. This group also observed interesting effects of tobacco smoke components in experiments using an epithelial cell line, derived from the human pancreatic duct [55]. In a scrape loading assay the vulnerated cells were loaded with Lucifer Yellow and the dye was detected in neighboring cells, the most obvious explanation of which is by way of intercellular channels, such as gap junctions. A distinct component of tobacco smoke, 1-methylanthracene with “bay-like structures”, inhibited the transfer of the dye. Another group compared the effect of tobacco smoke components with primarily heated tobacco [56]. Human airway epithelial cells, coronary artery endothelial cells, keratinocytes, and the above-mentioned rat liver epithelial cell line were studied using fluorescence redistribution after photobleaching; the so-called fluorescence recovery after photobleaching (FRAP) technique. In contrast to the previously described technique, all cells were loaded with a fluorescent dye and the neighboring cells of a determined cell were bleached using a laser beam. Interestingly, the heated tobacco did not inhibit GJIC between the tested human cells, whereas components released from burned tobacco at equivalent concentrations did inhibit the intercellular communication. An additional observation made in this same study was that, in contrast to burned tobacco, the heated tobacco did not affect the release of lactate dehydrogenase (LDH). This indicates that heated tobacco creates a lower level of cell toxicity than burned tobacco. So-called dye-coupling, or parachute assays, have been performed using dye-loaded donor cells and non-loaded acceptor cells [57]. This assay clearly demonstrated that the condensate of tobacco smoke or tobacco smoke-derived particles, respectively, clearly decreased the transfer of the fluorescent dye from donor to acceptor cells [58]. Cells derived from the epithelial cell line (WB-F344) of a rat liver were prepared as donor cells and loaded with a dye. A transfer of intracellular fluorescent calcein from donor cells to neighboring cells was determined. After subtraction of stained donor cells, the percentage of stained cells represented the proportion of GJIC. Calcien can enter the membranes of intact cells and minimize cellular stress [58]. Tobacco smoke-derived particles from cigarettes inhibited the dye transfer, and thus that of GJIC, and concentrations of approx. 0.02 mg/mL and above were measured. In tobacco smoke-derived particles, however, a half-maximal effective concentration of approx. 0.05 mg/mL was established. The authors assumed that a major impact of polycyclic aromatic hydrocarbons (PAHs) was responsible for the inhibition of the observed GJIC. Interestingly, arsenic disrupts the molecular structure of connexin 43 and interrupts GJIC, which might contribute to the toxicity and carcinogenicity of arsenic [59]. Cadmium or cadmium salts, respectively, also inhibit the gap junction-mediated communication between rat liver cells (cell line BRL 3A), as shown in a scrape loading assay [60]. Cadmium and GJIC were investigated using a parachute assay in prostate cells [61]. A low dose of CdCl_2_ stimulated cell proliferation, increased the expression of connexin 43, and reduced the dye transfer between donor and acceptor cells by half. Aromatic amines, such as aniline, toluidine, or naphthylamine, and others are important occupational toxic agents, for instance in the chemical industry, but they are also components of tobacco smoke, drugs, pesticides, hair tinting lotions, and other products [62]. So far, these aromatic amines have not been reported to represent strong risk factors for urinary bladder carcinomas on GJIC. Interestingly, catechins, as ingredients of green teas, have displayed positive effects in experiments using renal epithelium, which was treated with nitrosamines [63]. In this study the pre-treatment with a distinct catechin ameliorated the reduction of connexin 43 expression and GJIC. The authors suggest a “chemo preventive” effect of green tea. Another relevant player is lipopolysaccharide (LPS), which is part of tobacco smoke or is released during bacterial infections. LPS has effects on GJIC and the presence of connexins, e.g. down regulation of connexin 43 and GJIC in astrocytes [64,65].

## 7. Putting it Together

**Figure 4:**
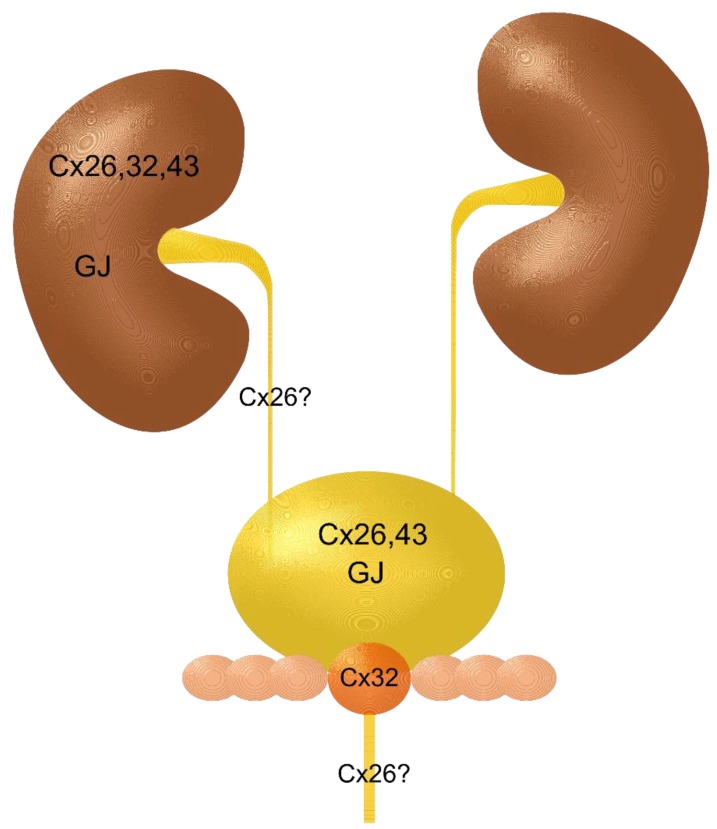
Schematic drawing of the urinary tract including kidneys, ureters, urinary bladder, prostate gland, seminal vesicles, and urethra. The drawing depicts the recently gathered data on connexins and gap junctions in the parts of the urinary tract.

Gap junctions have a crucial role in carcinogenesis in general, but especially in epithelia of the human urinary tract. Endogenous, but even, to a greater extent, exogenous, environmental factors trigger the epigenetic limb of pro-carcinogenic mechanisms, which often lead over years and decades to carcinomas (Figure 4). So far, as investigated and reported, the connexins 26, 32, and mostly connexin 43, play a relevant role (Figure 4). From the published data, a cautious conclusion can be drawn that connexin 26 is expressed and functionally active in the urothelium and the tubular epithelia. Connexin 32 was reported within the tubular and the prostate epithelia. Connexin 43 seems to be upregulated in the tubular epithelia and in the urothelium of the urinary bladder, if hyperplasia and carcinoma occurs. Data on GJIC and its modulation are available, but there remains a knowledge gap with regard to the descriptive morphology of gap junctions in the tissues of the human urinary tract. From the authors´ point of view, this central question has yet to be addressed. Gap junctional intercellular communication is without doubt involved in physiology, pathophysiology, carcinogenesis, and carcinoma growth and metabolism. However, the available data do not allow conclusive judgement as to whether connexins, connexons, and gap junctions promote or inhibit the development of carcinomas within the human urinary tract. It is not unlikely, that in the case of an established carcinoma, the GJIC is recruited from the cancer tissue to promote growth and vascularization. If that is the case, inhibiting approaches or the destruction of structural components could be beneficial to patients. Thus, GJIC in cancers of the urogenital tract remains a field wide open to research.

## 8. Concluding Remark

In conclusion, distinct connexins and related molecules, pannexins, play a role in tumorigenesis in urinary bladder cancer [14]. Since these connexins build gap junctions, their involvement in tumorigenesis is most likely. Studies of human urinary carcinoma should include freeze-fracture and freeze-fracture immune-labelling to elucidate the role of gap junctions in this type of tumor.
